# Comparative effects of oleoyl-estrone and a specific β_3_-adrenergic agonist (CL316, 243) on the expression of genes involved in energy metabolism of rat white adipose tissue

**DOI:** 10.1186/1743-7075-7-15

**Published:** 2010-02-25

**Authors:** Raquel Ferrer-Lorente, Cristina Cabot, José-Antonio Fernández-López, Marià Alemany

**Affiliations:** 1 Department of Nutrition and Food Science, Faculty of Biology, University of Barcelona, and CIBER Obesity and Nutrition, Institute of Health Carlos III, Barcelona, Spain

## Abstract

**Background:**

The combination of oleoyl-estrone (OE) and a selective β_3_-adrenergic agonist (B3A; CL316,243) treatment in rats results in a profound and rapid wasting of body reserves (lipid).

**Methods:**

In the present study we investigated the effect of OE (oral gavage) and/or B3A (subcutaneous constant infusion) administration for 10 days to overweight male rats, compared with controls, on three distinct white adipose tissue (WAT) sites: subcutaneous inguinal, retroperitoneal and epididymal. Tissue weight, DNA (and, from these values cellularity), cAMP content and the expression of several key energy handling metabolism and control genes were analyzed and computed in relation to the whole site mass.

**Results:**

Both OE and B3A significantly decreased WAT mass, with no loss of DNA (cell numbers). OE decreased and B3A increased cAMP. Gene expression patterns were markedly different for OE and B3A. OE tended to decrease expression of most genes studied, with no changes (versus controls) of lipolytic but decrease of lipogenic enzyme genes. The effects of B3A were widely different, with a generalized increase in the expression of most genes, including the adrenergic receptors, and, especially the uncoupling protein UCP1.

**Discussion:**

OE and B3A, elicit widely different responses in WAT gene expression, end producing similar effects, such as shrinking of WAT, loss of fat, maintenance of cell numbers. OE acted essentially on the balance of lipolysis-lipogenesis and the blocking of the uptake of substrates; its decrease of synthesis favouring lipolysis. B3A induced a shotgun increase in the expression of most regulatory systems in the adipocyte, an effect that in the end favoured again the loss of lipid; this barely selective increase probably produces inefficiency, which coupled with the increase in UCP1 expression may help WAT to waste energy through thermogenesis.

**Conclusions:**

There were considerable differences in the responses of the three WAT sites. OE in general lowered gene expression and stealthily induced a substrate imbalance. B3A increasing the expression of most genes enhanced energy waste through inefficiency rather than through specific pathway activation. There was not a synergistic effect between OE and B3A in WAT, but their combined action increased WAT energy waste.

## Background

White adipose tissue (WAT) is a fairly active agent in the regulation of the energy availability of the body [1], superseding its classic role of energy storage depot [2] and playing a role in the endocrine/paracrine and metabolic regulation of energy substrate utilization [3]. The main elements that WAT employs in this regulative role are: a) its large capability to store energy as triacylglycerols, depleting circulating levels [4], a function that extends in part to the removal of excess glucose [5] used for lipogenesis [6]; b) its ability to reverse the storage process, releasing fatty acids to the bloodstream through lipolysis [7]; c) the modulation of other (and self) tissues through the secretion of hormones and paracrine agents [8]; and d) the wasting of energy, a postulated role [9] traditionally attributed to brown adipose tissue, and insufficiently characterized in WAT.

Oleoyl-estrone (OE) is a signalling molecule from WAT that has been postulated as a ponderostat modifying agent [10], since its administration induces a severe wasting of the body fat reserves [11], reducing WAT lipogenesis and substrate uptake rather than elicit increases in lipolysis, increasing fatty acid output [12]. This process is in part mediated, at least in liver, by SREBP1c [13], under conditions in which glycolysis is not completely blocked irrespective of a large availability of fatty acids [14]. Notwithstanding, basically the OE mechanism of action remains largely unknown.

The combined administration of a specific β_3_-adrenergic agonist with OE results in an assumedly synergistic effect of both agents inducing a massive WAT wasting, but nevertheless maintaining the circulating levels of the main metabolic indicators within the normalcy range [15]. OE reduces WAT mass at the expense of a decrease in energy intake with maintained thermogenesis [16,17], whilst most β_3_-adrenergic agonists elicit a massive lipolysis to fuel an increased thermogenesis, but barely affect food intake [18], and tend to show limited effects because of downregulation of the receptors [19].

The discovery of β_3_-adrenergic receptors in brown and white adipose tissue [20] generated an enormous research effort to obtain agonists for these receptors that could elicit an increase in thermogenesis without the dangerous secondary effects of unspecific β-adrenergic stimulation [21,22]. A large number of compounds were obtained [22] but none was finally commercialized because of the short-time span of their efficacy and the rapid down-regulation of the receptors [20]. There is a number of β_3_-adrenergic agonists available that show very little superposition in their effects with β_1 _or β_2 _receptors. We had previously used CL316,243 [15] in a previous gross-energy study, in combination of OE, and found that this combination deeply affected the body energy reserves [15]. As a continuation, the purpose of this study is to check how WAT responds to the double challenge, determining whether there is a common sharing of pathways for WAT energy disposal, or their actions upon the tissue follow separate strategies. Finally, we also wanted to know whether there is (or not) a synergistic effect favoring the depletion of body fat when using the combined slimming agents approach.

## Methods

### Animals and experimental setup

Male Wistar rats (Harlan-Interfauna, Sant Feliu de Codines, Spain) of 45 days were used. The rats were maintained under standard conditions (21-22°C, 60-50 % relative humidity, and 12 h light/dark cycle: on from 08.00) in three-rat cages, and were fed for five weeks a simplified cafeteria diet [20]*ad libitum*. As previously described [23], at the end of this phase the rats were already overweight; the animals were re-conditioned to eat standard pellet diet as sole food during an additional week (maintenance chow, Panlab, Barcelona, Spain).

Four groups of six animals, initially weighing 360-380 g, were randomly selected: a) Controls; b) oleoyl-estrone: OE; c) β_3_-adrenergic agonist (B3A) CL316,243: B3A; and d) oleoyl-estrone and β_3_-adrenergic agonist: OE+B3A. Groups B3A and OE+B3A were implanted on day 0 subcutaneously in the back with an osmotic Alzet minupump (type 2002, Alzet, Palo Alto CA, USA) under isoflurane anaesthesia; the minipumps were loaded with B3A, the β_3_-adrenergic agonist CL316,243 [24](Sigma, St Louis, MO USA) dissolved in saline. The minipumps released B3A at a rate of 0.5 μl/h in the subcutaneous space, at a dose of 1 mg/kg and day. The rats in the OE and OE+B3A groups received a gavage containing OE (OED, Barcelona, Spain) in 0.2 ml sunflower oil, at a daily dose of 10 μmol/kg. The gavage of rats in the control and B3A groups contained only oil. The rats were given the gavages at the beginning of the light cycle, and were maintained under standard housing conditions, with full access to water and food pellets; their weight and food consumption were recorded daily. The treatments continued for 10 days.

At the end of the experiment, the rats were killed by decapitation. The rats were dissected and samples of inguinal, retroperitoneal and epididymal WAT were rapidly taken, frozen in liquid N and weighed. Then, the three WAT pads (inguinal subcutaneous, retroperitoneal and epididymal) were completely dissected, blotted and weighed in order to obtain the total mass of each WAT site. Tissue samples were maintained at -80°C until used

The experimental setup and procedures were approved by the Ethics Committee of the University of Barcelona. All animal handling procedures were carried out following the guidelines established by the EU, and the Spanish and Catalan Governments.

### Nucleic acid analyses, cellularity

Tissue samples were used for the estimation of total DNA, using a standard fluorimetric method with 3,5-diaminobenzoic acid (Sigma, St Louis MO USA) and bovine DNA (Sigma) as standard [25]. Tissue DNA content allowed the calculation of the number of cells per g of tissue and in the whole tissues sampled, based on the assumption that the DNA content per cell is constant in mammals; here we used the genomic DNA size data [26] for somatic rat cells (5.6 pg/cell). Mean cell mass was estimated from the number of cells and the tissue weight.

Total tissue RNA was extracted using the Tripure reagent (Roche Applied Science, Indianapolis IN USA), and were quantified in a ND-100 spectrophotometer (Nanodrop Technologies, Wilmington DE USA).

### Semiquantitative RT-PCR analysis of gene expression

RNA samples were reverse transcribed using the MMLV reverse transcriptase (Promega, Madison, W I USA) and oligo-dT primers. Real-time PCR (RT-PCR) amplification was carried out using 0.010 mL amplification mixtures containing Power SYBR Green PCR Master Mix (Applied Biosystems, Foster City, CA USA), equivalent to 8 ng of reverse-transcribed RNA and 300 nM primers. Reactions were run on an ABI PRISM 7900 HT detection system (Applied Biosystems) using a fluorescent threshold manually set to OD 0.500 for all runs. W e analyzed the expression of the genes listed in the Additional File 1 Table S1, which includes the list of primers used.

A semiquantitative approach for the ultimate estimation of the number of copies of each expressed gene mRNAs per tissue weight was used as previously described [27]. ARBP was used as charge control gene in all samples. All primers were selected and optimized to obtain values of r ≥ 0.98, resulting in an efficiency range of 93-104 %.

### cAMP analysis

Frozen tissue samples were homogenized in chilled perchloric acid 30 g/L [28] using a Polytron tissue disruptor (ultraturrax, Kinematica, Luzern, Switzerland); after neutralization with 2N NaOH, supernatants were used for a cAMP binding assay using a specific kit (TRK432, GE Healthcare, Little Chalfont, UK). Results for cAMP were expressed in amol/cell; cAMP data, as well as specific gene mRNA content in the tissue were expressed as absolute data of content in the whole of the specific fat pad, as a way to specifically discriminate the global WAT site response to the stimuli regardless of cell size or number, parameters that may be also influenced by the treatments [16,27].

### Statistical analysis

In addition to the three sites studied, a fourth group of data was included, corresponding to the sum of the three sites. The data were obtained tabulating the experimental results for each animal. Statistical two-way ANOVA analyses was carried out using the Prism 5 program package (GraphPad Software, La Jolla, CA USA), with a limit of significance of P < 0.05. Spot comparisons between groups were done using the unpaired Student's *t *test.

## Results

### Body weight, tissue cellularity and cAMP levels

**Figure 1:**
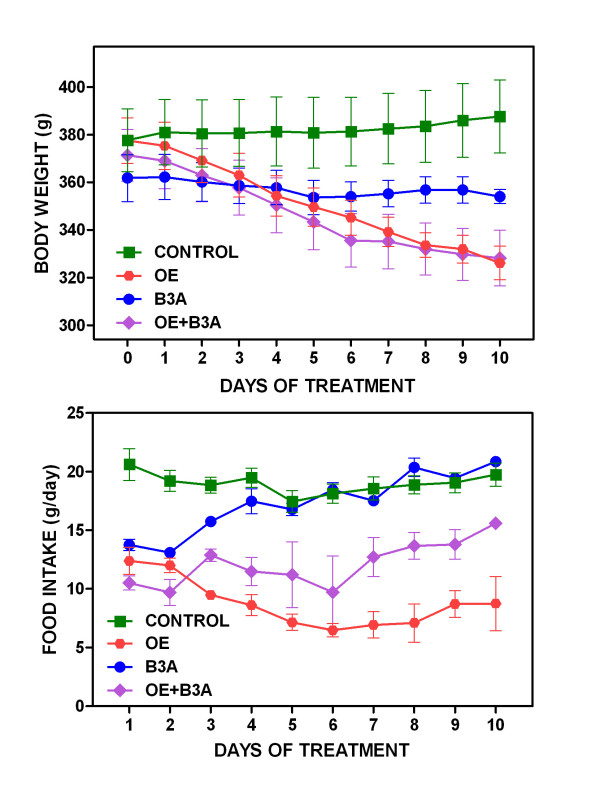
**Body weight and food intake changes in overweight rats treated with OE and/or B3A during the 10-day study**. The data are the mean ± sem of six different animals.

Figure 1 shows the changes in body weight and food intake elicited by the 10-day pharmacologic treatment. Control rats increased their weight by a 2.4 ± 0.7 %; OE rats lost 13.7 ± 1.4 %, B3A lost 2.2 ± 2.5 % and OE+B3A lost 11.7 ± 1.0 % in 10 days; the effect of OE was significant (P < 0.0001) but not that of B3A; there was a significant (P = 0.0468) interaction between both agents (two-way ANOVA). Food intake in controls was a mean 18.6 ± 0.8 g/day, in the B3A group it was slightly decreased (16.3 ± 1.1 g/day), and showed even lower values in the OE (8.4 ± 0.6 g/day) and combined OE+B3A (12.8 ± 0.9 g/d) groups; the effects of OE on food intake were statistically significant (P < 0.0001), but not those of B3A (two-way ANOVA).

Table 1 presents the data on total weight and cellularity of the three WAT sites studied. Both agents induced marked reductions in weight, with significant effects of both OE and B3A. These effects were extended to the sum of the weight of these WAT sites, resulting in overall losses in the range of 60 % in the combined WAT mass for the OE+B3A group.

**Table 1:** Weight/cell content of three main WAT sites of overweight male rats treated for 10 days with combined OE and B3A

parameter	units	control	OE	B3A	OE+B3A	P- OE	P-B3A	P-int
inguinal subcutaneous WAT weight	g	4.30 ± 0.28	3.49 ± 0.31	3.04 ± 0.56	2.87 ± 0.54	NS	0.0339	NS

epidydimal WAT weight	g	10.37 ± 0.92	8.59 ± 0.53	6.47 ± 0.61	5.05 ± 0.90	0.0481	0.0000	NS

retroperitoneal WAT weight	g	11.81 ± 1.24	8.19 ± 0.69	3.20 ± 0.64	3.26 ± 0.64	0.0408	0.0000	0.0472

combined WAT weight	g	26.48 ± 3.07	20.27 ± 1.31	12.71 ± 1.75	10.53 ± 1.39	0.0497	0.0000	NS

inguinal subcutaneous WAT cellularity	10^6 ^cells	618 ± 74	549 ± 55	549 ± 29	553 ± 53	NS	NS	NS

inguinal subcutaneous WAT cell mass	ng	7.66 ± 1.24	6.83 ± 0.71	6.21 ± 0.45	4.24 ± 0.61	NS	0.0213	NS

epidydimal WAT cellularity	10^6 ^cells	359 ± 45	439 ± 32	459 ± 75	385 ± 16	NS	NS	0.0039

epidydimal WAT cell mass	ng	24.9 ± 1.3	22.4 ± 1.9	12.9 ± 1.1	12.5 ± 0.8	NS	0.0000	NS

retroperitoneal WAT cellularity	10^6 ^cells	431 ± 70	340 ± 69	372 ± 11	377 ± 73	NS	NS	NS

retroperitoneal WAT cell mass	ng	28.1 ± 1.8	29.0 ± 6.0	7.54 ± 0.33	8.29 ± 0.90	NS	0.0000	NS

The values are the mean ± sem of 6 different animals. Statistical significance of the diferences (2-way ANOVA): P-OE/PB3A represents the P values for OE/B3A inducing a significant effect on the given parameter; P-int indicates the significance of the interaction between both agonists. NS = p > 0.05.

The estimated number of cells was not affected in any of the three WAT sites by either treatment; however, the mean cell weight decreased in all (as did their overall mass), the effects of B3A being significant for all sites and those of OE for none of them.

Table 2 shows the cAMP content of WAT after treatment with OE and B3A. OE effects were limited: the only significant values were decreases in epididymal WAT (only when expressed per cell unit), and in the total content of retroperitoneal and combined WAT. In contrast, B3A increased the cAMP levels of epididymal and combined WAT sites. No significant interactions were observed.

**Table 2:** cAMP tissue/cell content in three main WAT sites of overweight male rats treated for 10 days with combined OE and B3A

organ/tissue	units	control	OE	B3A	OE+B3A	P-OE	P-B3A	P-int
inguinal subcutaneous WAT	amol/cell	1.91 ± 0.42	1.92 ± 0.30	2.37 ± 0.37	1.88 ± 0.59	NS	NS	NS
	
	nmol	0.93 ± 0.11	0.73 ± 0.09	1.14 ± 0.22	0.97 ± 0.22	NS	NS	NS

epididymal WAT	amol/cell	3.74 ± 0.57	2.72 ± 0.20	5.92 ± 0.39	4.71 ± 0.36	0.0117	0.0000	NS
	
	nmol	1.35 ± 0.17	1.09 ± 0.10	2.61 ± 0.31	2.06 ± 0.31	NS	0.0002	NS

retroperitoneal WAT	amol/cell	6.02 ± 0.42	4.53 ± 0.86	4.64 ± 0.20	3.83 ± 0.75	NS	NS	NS
	
	nmol	2.52 ± 0.30	1.31 ± 0.26	1.75 ± 0.23	1.61 ± 0.27	0.0197	NS	NS

combined WAT	nmol	4.79 ± 0.43	3.49 ± 0.35	5.49 ± 0.46	4.66 ± 0.67	0.0427	0.0000	NS

The values are the mean ± sem of 6 different animals. Statistical significance of the diferences (2-way ANOVA): P-OE/PB3A represents the P values for OE/B3A inducing a significant effect on the given parameter; P-int indicates the significance of the interaction between both agonists. NS = p > 0.05.

### Tissue gene expressions in individual WAT sites

**Figure 2:**
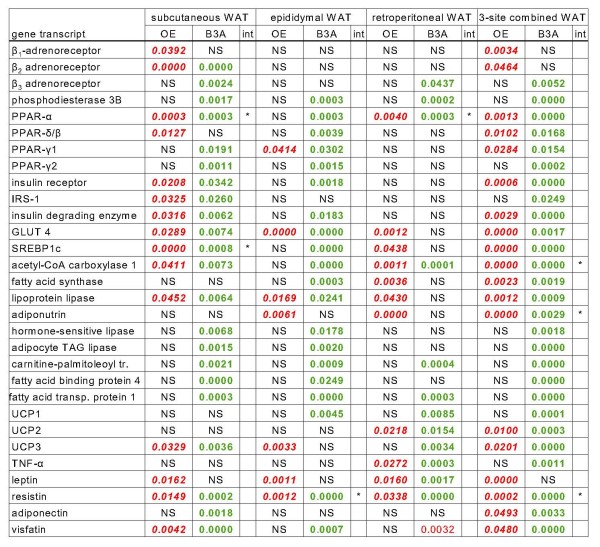
**P values for the statistical significance of the effects of OE and B3A on the gene expression of lipid metabolism -related enzymes and factors in the main sites of WAT of male overweight rats treated with OE and a B3A (two-way ANOVA)**. Data in bold red italics represent significant decreases in the combined specific mRNA content in the given WAT site (and, in the case of "combined WAT" in the sum of all three sites) compared with controls receiving vehicle only. Data in bold green represent significant increases. Non-significant effects (NS) are in black. An asterisk * in the interaction (int) column denotes a significant (P < 0.05) interaction between OE and B3A effects.

The tissue levels of specific mRNAs for the genes studied are presented in the Additional File 1, Tables S2, S3, S4 and S5 for, respectively, subcutaneous, epididymal, retroperitoneal and combined WAT sites. The statistical analysis of the differences in expression of the genes for these WAT sites is presented in Figure 2.

In subcutaneous WAT, OE decreased the expression of β_1 _and β_2_-adrenoceptor genes, as well as PPAR-α and PPAR-β/δ. Insulin signaling was decreased: lower expressions of insulin receptor (but also its degrading enzyme), IRS-1, and GLUT-4 genes. A similar decrease in expression was observed for lipogenic agents SRBP1c, and acetyl-CoA carboxylase genes. Lipoprotein lipase, UCP3 and the cytokines leptin, resistin and visfatin expressions were also decreased. The effects of B3A were markedly different, since in this case all gene expressions studied were increased with respect to controls, with the (non statistically significant) exceptions of the β_1_-adrenal receptor, PPAR-β/δ, fatty acid synthase, adiponutrin, UCP1, UCP2, and the cytokines leptin and TNFα.

The pattern of gene expression decreases elicited by OE was less marked in epididymal than in subcutaneous WAT, with only significant changes in PPARγ_1_, GLUT 4, lipoprotein lipase, adiponutrin, UCP3, leptin and resistin genes. However, the increases in gene expression induced by B3A were fully comparable to those of subcutaneous WAT, the only differences being: no significant effects on β-adrenal receptors, IRS-1, UCP3 and adiponectin genes, but significant differences in fatty acid synthase and UCP1 gene expressions.

In retroperitoneal WAT, OE effects on gene expression were again different from the other two WAT sites. OE induced significant decreases in the gene expression of PPAR-α, GLUT4 and a number of lipid-metabolism related genes: SREBP1c, acetyl-CoA carboxylase 1, fatty acid synthase, lipoprotein lipase and adiponutrin. OE also induced decreases in the expression of UCP2 and the adipokines TNFα, leptin and resistin. The administration of B3A (alone or combined with OE) induced much less change in gene. expressions than in subcutaneous and retroperitoneal WAT. In this case B3A induced a decrease in the expression of the visfatin gene and increases in the β_3_-adrenergic receptor, phosphodiesterase 3B, PPAR-α, acetyl-CoA carboxylase, carnitine palmitoleoyl-transferase, fatty acid transporting protein 1, the UCPs, TNFα, leptin and resistin.

### Tissue gene expressions in combined WAT

When the data for gene specific transcripts present in the combined WAT sites were analyzed, the results became perhaps more clear: OE induced decreases in the expression of most genes studied, with the exceptions of β_3_-adrenergic receptor and phosphodiesterase 3B, PPARγ_2_, and IRS-1 as critical signaling and signal-transduction agents. There is a second group in which OE overall did not change significantly the expression of the WAT genes, all related with lipolysis and fatty acid import/transport: hormone sensitive lipase, adipocyte TAG lipase, carnitine palmitoleoyl-transferase, and the fatty acid binding and transporting protein genes, as well as the markedly apoptotic-inducing adipokine TNF-α gene; these genes maintained mRNA strengths comparable with those of controls despite WAT size reduction, since they showed no significant differences in absolute terms. The effects of B3A were markedly uniform: all genes studied increased their expression versus controls with the sole exception of three: β_1_- and β_2_-adrenal receptors and leptin.

## Discussion

OE effects on the overall energy economy [11,16] are often more marked than in the actual changes in plasma metabolite [11,29] levels or the spectrum of gene expression changes [12-14]. This includes WAT, which in the end provides most of the energy wasted to maintain energy expenditure under a decreased energy intake also consequence of OE treatment [30]. In the present study we have confirmed the considerable differences that the main WAT sites present with respect to their response to OE [31] or to other hormonal [32] or pharmacological agents [33].

It has been postulated that OE decreases WAT energy content by deregulating the equilibrium between lipogenesis (and triacylglycerol synthesis) and lipolysis [12,13]. In spite of marked differences between WAT sites, the cells of rats treated with OE were shrunk by the loss of fat; their decreased cAMP levels agreed with a generalized depression of lipogenesis. The lipolytic paths were not (or less-) affected, and fatty acid transport (both in, out of the cell, as well as into the mitochondria) was also decreased. It has been found previously (and the present result confirm -at least for WAT-) that OE does not induce marked changes in the expression of adrenergic receptor genes [28].

OE seems to induce a lethargic metabolic transformation of WAT, with generalized inhibition of metabolic activity, loss of fat and decrease in overall size [12] diminished hormonal and cytokine [31] signaling, and a more marked inhibition of the entry of energy substrates. Nevertheless, OE induces a marked loss of fat maintaining thermogenesis and the energy homeostasis exemplified by metabolite levels in plasma [29].

The β_3_-adrenergic agonists were initially designed as brown adipose tissue stimulators -i.e. thermogenic agents- [34] because of their abundance in this tissue. However, the presence of β_3_-adrenergic receptors in WAT [35] independently of a wide site-related variation [36], provides an additional foothold for their overall lipolytic and thermogenic effects; the increased cAMP levels found in WAT in B3A rats agree with their presence and functionality in the tissue. Curiously, B3A did not down-regulate the gene expression of the very receptors on which it acts, the β_3 _in contrast with the known problem of most β_3_-receptor agonists that rapidly lose effectiveness because of receptor downregulation [19]. In the present experiment, B3A has acted continuously (constant injection) for 10 days, inducing a severe wasting of WAT, but β_3_-adrenoceptors were downregulated by OE and not by B3A. This suggests that the generalized increase in gene expression induced by B3A is not only a consequence of its effects on its receptors, but possibly an effect also mediated through other hormonal or metabolic signal(s) affecting the whole animal but which effects were patently observed in WAT.

When the data for cAMP were calculated as nmol/g of tissue (a rough measure of its actual concentration in the tissue fluids) and correlated with the loss of weight experienced by the WAT site, irrespective of the treatment (i.e. controls, OE, B3A and their combination), we obtained a significant relationship between both parameters (r^2 ^= 0.813; p < 0.0001). There were no differences between OE and B3A, which suggests that the loss of tissue weight (i.e. fat) was a closely direct correlate of the functional concentration of cAMP in the tissue. The increase in cAMP also elicited a marked increase in the expression of the phosphodiesterase gene (about twice than that of combined β receptors). Thus, fat mobilization was largely induced via cAMP cascade, and the mechanisms to control this increase were appropriately set in place both for OE and B3A.

However, the almost universal increase in gene expression observed in rats treated with B3A suggests that probably its main effect is not the simple mobilization of fat, which proceeds at a pace comparable to that of OE -in fact carried out with much less fanfare- since there is a parallel stimulation of both lipolytic and lipogenic paths; glucose entry is probably enhanced (GLUT4, insulin receptor gene expressions) to fuel lipogenesis (SREBP1c, acetyl-CoA carboxylase, fatty acid synthase gene expressions), which combined with increased lipoprotein lipase and fatty acid transporters must provide a sizeable entry of substrates from the plasma. The problem is that lipolysis (hormone-sensitive lipase, adipocyte triacylglycerol lipase) and cAMP were also enhanced. All PPAR gene expressions, both lipogenic [37] and lipolytic [38] were also increased. Even contrary signals such as adiponectin and resistin were probably stimulated. The effectiveness of a contradictory stimulation of opposed pathways results in itself in a relative waste of energy, in a Penelope's Web way.

The limited overall effects of B3A on body size may in part reflect this disorganized process: adrenergic stimulation usually induces the loss of body weight through fat mobilization, but β_2 _agonists have been observed to increase body weight by modifying the accumulation of protein in muscle (e.g. clembuterol, [39]), an effect in part shared by other β_3 _agonists [40].

**Figure 3:**
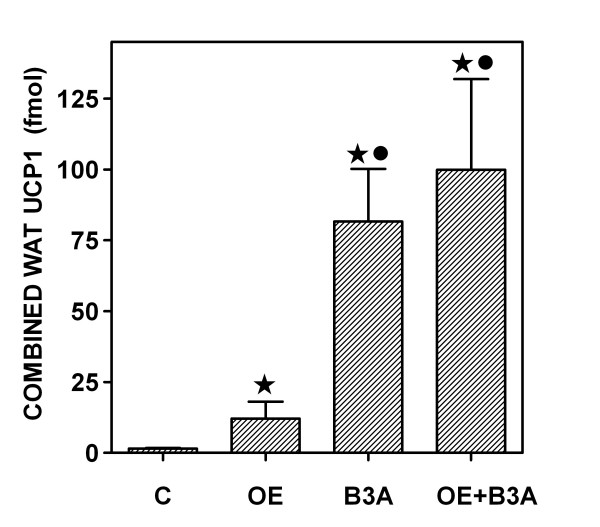
**Combined WAT (i.e. subcutaneous inguinal, epididymal and retroperitoneal) content of UCP1 gene mRNA in overweight rats treated with OE and/or B3A for 10 days**. The data are the mean ± sem of six different animals. Statistical significance of the differences between groups: asterisk = p < 0.05 versus controls; black circle = p < 0.05 OE versus B3A and B3A+OE; no significant differences were found between B3A and B3A+OE.

The considerable increase in the expression of UCP1 gene due to B3A stimulation (56× alone or 68× combined with OE) (Figure 3), yields an amount of UCP1 mRNA in the range of 15 % of that found in the interscapular brown adipose tissue mass of an adult male rat (unpublished data). This may represent that WAT plays a role in the marked increase of thermogenesis elicited by B3A, both in this same experimental model [15] and others [34]. The "browning" of WAT was less affected by OE, which, in turn, decreased the gene expression of UCP2 and UCP3, which function seems more related with transport and regulation [41] than pure thermogenesis, reserved to UCP1 [42]. The increase in UCP1 mRNA content represents an even higher concentration per unit of tissue weight/per cell, since the WAT mass shrunk considerably compared with controls. In spite of OE not changing (or relatively decreasing) UCP1 gene expression in BAT [43], its more limited effect on WAT (8× increase) could not be linked with adrenergic stimulation (low cAMP levels), and agrees with the marked differences in energy metabolism observed in brown adipose tissue [38] compared with WAT [44]

There are reports linking the conversion of white to brown adipocytes as a consequence of continued catecholaminergic stimulation [45]; in the present study, however, subcutaneous WAT did not respond to these same stimuli as did the other WAT masses. The analysis of WAT metabolism and overall function as an organ [46] or as a cluster of similar organs, is thoroughly complicated by their diversity in substrate processing, response to hormonal stimuli and their implication in the regulation of energy homeostasis [47]. A simple look at Figure 2 shows that subcutaneous WAT responds more easily to OE and B3A than the retroperitoneal, and the differences between epididymal and retroperitoneal (they are in part anatomically connected in the rat visceral cavity) are also considerable.

It is unclear whether the marked alterations in gene expression observed here translate into changes of their corresponding proteins, but at least they are a fair indicator of the processes that are activated, and the loss of fat (i.e. lower cell size and lower WAT site sizes) is a proven consequence that adequately fits with this explanation.

In sum, it is rather surprising that two different agents, with different mechanisms of action, that elicit widely opposed responses in WAT gene expression (OE stealthy but focused in the substrate imbalance; B3A loud and contrasting, resulting in handling-derived energy waste) end producing a very similar effect: shrinking of WAT, loss of fat, maintenance of cell numbers (i.e. practically no apoptosis). In addition, at the level of the whole animal, their effects are additive: OE decreases appetite, B3A increases thermogenesis, both waste WAT: the result is an accelerated loss of energy of up to 2.5 % of body energy per day in overweight rats [15].

## Conclusions

OE and B3A, elicit widely different responses in WAT gene expression on the three sites studied. Both drugs final effects were similar: shrinking of WAT size because of loss of fat but maintenance of cell numbers. OE acted essentially on the balance of lipolysis-lipogenesis and the blocking of the uptake of substrates; essentially hampering synthesis, and thus favoring lipolysis indirectly. B3A induced a shotgun increase in the expression of most regulatory systems in the adipocyte, an effect that in the end favored again the loss of lipid, probably due to the loss of metabolic coordination; this barely selective increase probably produces inefficiency. The waste of energy induced by B3A was compounded by the increase in UCP1 gene expression, since UCP1 provides thermogenic capability to adipocytes, thus increasing the energy waste by WAT itself as a contribution to the increase in overall thermogenesis induced by B3A.

Thus, OE in general lowered gene expression and stealthily induced a substrate imbalance. B3A increasing the expression of most genes enhanced energy waste through inefficiency rather than through specific pathway activation. There was not a synergistic effect between OE and B3A in WAT, but their combined action increased WAT energy waste.

## List of Abbreviations

WAT: white adipose tissue; UCP1: uncoupling protein 1; OE: oleoyl-estrone; B3A: CL316,243, a β_3_-adrenergic agonist.

## Competing interests

The authors declare that they have no competing interests.

## Authors' contributions

RFL did most of the experimental work, obtaining and refining the results. She also did the statistical analysis. CC intervened in a number of experiments, and in the gathering of data. CC and JAFL established the protocols, directed the work and analyzed and refined the results, completing the statistical analysis. MA established the final conclusions, and wrote the manuscript. The text was reviewed and agreed to by all Authors.

## Supplementary Material

Additional file 1 **WAT GENE EXPRESSIONS**. content: Table S1 - Primers used in the analysis of gene expression. Table S2. Whole inguinal subcutaneous WAT content of specific gene mRNAs of rats overweight male treated 10 days with OE and B3A. Table S3. Whole epididymal WAT content of specific gene mRNAs of rats overweight male treated 10 days with OE and B3A. Table S4. Whole retroperitoneal WAT content of specific gene mRNAs of rats overweight male treated 10 days with OE and B3A. Table S5. Combined inguinal subcutaneous. epididymal and retroperitoneal WAT content of specific gene mRNAs of rats overweight male treated 10 days with OE and B3A. Click here for file

## References

[B1] Klaus S Adipose tissue as a regulator of energy balance Curr Drug Targets 2004 5 241 250 10.2174/1389450043490523 15058310

[B2] Badman MK Flier JS The adipocyte as an active participant in energy balance and metabolism Gastroenterol 2007 132 2103 2115 10.1053/j.gastro.2007.03.058 17498506

[B3] Wozniak SE Gee LL Wachtel MS Frezza EE Adipose tissue: the new endocrine organ? A review article Digest Dis Sci 2009 54 1847 1856 10.1007/s10620-008-0585-3 19052866

[B4] Mårin P Rebuffé-Scrive M Björntorp P Uptake of triglyceride fatty acids in adipose tissue in vivo in man Eur J Clin Invest 1990 20 158 165 2112480 10.1111/j.1365-2362.1990.tb02263.x

[B5] Mårin P Rebuffé-Scrive M Smith U Björntorp P Glucose uptake in human adipose tissue Metabolism 1987 36 1154 1160 10.1016/0026-0495(87)90242-3 3316924

[B6] Baquer NZ Sinclair M Kunjara S Yadav UCS Mclean P Regulation of glucose utilization and lipogenesis in adipose tissue of diabetic and fat fed animals: Effects of insulin and manganese J Bosci 2003 28 215 221 10.1007/BF02706221 12711814

[B7] Langin D Control of fatty acid and glycerol release in adipose tissue lipolysis Comp Rend Biol 2006 329 598 607 10.1016/j.crvi.2005.10.008 16860278

[B8] Pond CM Paracrine interactions of mammalian adipose tissue J Exp Zool 2003 295 99 110 10.1002/jez.a.10215 12506408

[B9] Granneman JG Burnazi M Zhu Z Schwamb LA White adipose tissue contributes to UCP1-independent thermogenesis Am J Physiol 2003 285 E1230 E1236 10.1152/ajpendo.00197.2003 12954594

[B10] Adán C Cabot C Esteve M Grasa MM Masanés R Vilà R Estruch J Fernández-López JA Remesar X Alemany M Oleoyl-estrone treatment affects the ponderostat setting differently in lean and obese Zucker rats Int J Obesity 1999 22 366 373 10.1038/sj.ijo.0800828 10340814

[B11] Grasa MM Cabot C Esteve M Yubero P Masanés RM Blay M Vilà R López-Martí J Fernández-López JA Remesar X Alemany M Daily oral oleoyl-estrone gavage induces a dose-dependent loss of fat in Wistar rats Obes Res 2001 9 202 209 10.1038/oby.2001.22 11323446

[B12] Romero MM Fernández-López JA Esteve M Alemany M Site-related white adipose tissue lipid-handling response to oleoyl-estrone treatment in overweight male rats Eur J Nutr 2009 48 291 299 10.1007/s00394-009-0013-2 19326039

[B13] Serrano M Grasa MM Janer G Fernández-López JA Alemany M Oleoyl-estrone enhances hepatic lipogenesis in adrenalectomized rats treated with corticosterone through modulation of SREBP1c expression J Steroid Biochem Mol Biol 2009 117 15 22 10.1016/j.jsbmb.2009.06.003 19545626

[B14] Romero MM Fernández-López JA Alemany M Esteve M Gene expression modulation of liver energy metabolism by oleoyl-estrone in overweight rats Biosci Reports 2010 30 81 89 10.1042/BSR20080182 19275765

[B15] Ferrer-Lorente R Cabot C Fernández-López JA Alemany M Combined effects of oleoyl-estrone and a β_3_-adrenergic agonist (CL316,243) on lipid stores of diet-induced overweight male Wistar rats Life Sci 2005 77 2051 2058 10.1016/j.lfs.2005.04.008 15935402

[B16] Balada F Sanchis D Grasa MM Virgili J Estruch J Fernández-López JA Remesar X Alemany M Effect of the slimming agent oleoyl-estrone in liposomes on the body weight of Zucker obese rats Int J Obesity 1997 21 789 895 10.1038/sj.ijo.0800475 9376892

[B17] Cabot C Grasa MM Fernández-López JA Alemany M Oleoyl-estrone treatment reduces the volume of white adipose tissue cells in the rat J Physiol Biochem 2000 56 369 370 10.1007/BF03179805 11321531

[B18] Atgié C Faintrenie G Carpéné C Bukowiecki LJ Géloën A Effects of chronic treatment with noradrenaline or a specific β_3_-adrenergic agonist, CL 316,243, on energy expenditure and epididymal adipocyte lipolytic activity in rat Comp Biochem Physiol A 1998 119A 629 636 10.1016/s1095-6433(97)00476-5 11249012

[B19] Lafontan M Berlan M Fat cell adrenergic receptors and the control of white and brown fat cell function J Lipid Res 1993 34 1057 1091 8371057

[B20] Muzzin P Revelli JP Kuhne F Gocayne JD McCombie WR Venter JC Giacobino JP Fraser CM An adipose tissue-specific β-adrenergic receptor. Molecular cloning and down-regulation in obesity J Biol Chem 1991 266 24053 24058 1721063

[B21] Lowell BB Flier JS The potential significance of β_3 _adrenergic receptors J Clin Invest 1995 95 923 10.1172/JCI117798 7883990 PMC441421

[B22] Fernández-López JA Remesar X Foz M Alemany M Pharmacological approaches for the treatment of obesity Drugs 2002 62 915 944 10.2165/00003495-200262060-00005 11929339

[B23] Ferrer-Lorente R Cabot C Fernández-López JA Remesar X Alemany M Effects of oleoyl-estrone with dexfenfluramine, sibutramine or phentermine on overweight rats Eur J Pharmacol 2005 513 243 248 10.1016/j.ejphar.2005.02.044 15862807

[B24] Yoshida T Umekawa T Sakane N Yoshimoto K Kondo M Effect of CL316,243, a highly specific β_3_-adrenoceptor agonist, on sympathetic nervous system activity in mice Metabolism 1996 45 787 791 10.1016/S0026-0495(96)90147-X 8637456

[B25] Vytasek RA A sensitive fluorometric assay for the determination of DNA Anal Biochem 1982 120 243 248 10.1016/0003-2697(82)90342-6 6283935

[B26] Rat Genome Sequencing Project Consortium Genome sequence of the Brown Norway rat yiels insights into mammalian evolution Nature 2004 428 493 521 10.1038/nature02426 15057822

[B27] Romero MM Grasa MM Fernández-López JA Esteve M Alemany M Semiquantitative RT-PCR measurement of gene expression in rat tissues including a correction for varying cell size and number Nutr Metab 2007 4 26 10.1186/1743-7075-4-26 PMC2217546 18039356

[B28] Cabot C Grasa MM Fernández-López JA Remesar X Alemany M Effect of oleoyl-estrone treatment on the expression of β_1_- β_2_- and β_3_-adrenoreceptors in rat adipose tissues Mol Cell Biochem 2001 221 109 115 10.1023/A:1010965527221 11506172

[B29] Sanchis D Adán C Ardévol A Grasa MM Cabot C Balada F Vilà R Estruch J Puerta ML Fernández-López JA Remesar X Alemany M Short-term treatment with oleoyl-oestrone in liposomes (Merlin-2) strongly reduces the expression of the *ob *gene in young rats Biochem J 1997 326 357 360 9291105 10.1042/bj3260357 PMC1218678

[B30] Sanchis D Balada F Picó C Grasa MM Virgili J Farrerons C Palou A Fernández-López JA Remesar X Alemany M Rats receiving the slimming agent oleoyl-estrone in liposomes (Merlin-2) decrease food intake but maintain thermogenesis Arch Physiol Biochem 1997 105 663 672 10.1076/apab.105.7.663.11391 9693713

[B31] Romero MM Fernández-López JA Esteve M Alemany M Different modulation of adipocytokine expression in four main white adipose tissue sites in the rat: mesenteric, perigonadal, retroperitoneal and subcutaneous J Cardiovasc Diabetol 2009 8 42 10.1186/1475-2840-8-42 PMC3224727 19642981

[B32] Prunet-Marcassus B Cousin B Caton D André M Pénicaud L Casteilla L From heterogeneity to plasticity in adipose tissues: Site-specific differences Exp Cell Res 2006 312 727 736 10.1016/j.yexcr.2005.11.021 16386732

[B33] BowenW P Flint DJ Vernon RG Regional and interspecific differences in the ligand binding properties of beta-adrenergic receptors of individual white adipose tissue depots in the sheep and rat Biochem Pharmacol 1992 44 681 686 10.1016/0006-2952(92)90403-6 1324682

[B34] Himms-Hagen J Cui JY Danforth E Taatjes DJ Lang SS WATers BL Claus TH Effect of Cl-316,243, a thermogenic β_3_-agonist, on energy- balance and brown and white adipose tissues in rats Am J Physiol 1994 266 R1371 R1382 7910436 10.1152/ajpregu.1994.266.4.R1371

[B35] Revelli JP Muzzin P Paoloni A Moinat M Giacobino JP Expression of the β_3_-adrenergic receptor in human white adipose tissue J Mol Endocrinol 1993 10 193 197 10.1677/jme.0.0100193 8387311

[B36] Krief S Lönnqvist F Raimbault S Baude B van Spronsen A Arner P Strosberg AD Ricquier D Emorine LJ Tissue distribution of β_3_-adrenergic receptor mRNA in man J Clin Invest 1993 91 344 349 10.1172/JCI116191 8380813 PMC330032

[B37] Knight BL Hebbachi A Hauton D Brown AM Wiggins D Patel DD Gibbons GF A role for PPARα in the control of SREBP activity and lipid synthesis in the liver Biochem J 2005 389 413 421 10.1042/BJ20041896 15777286 PMC1175119

[B38] Festuccia WT Laplante M Berthiaume M Gélinas Y Deshaies Y PPARα agonism increases rat adipose tissue lipolysis, expression of glyceride lipases, and the response of lipolysis to hormonal control Diabetologia 2006 49 2427 2436 10.1007/s00125-006-0336-y 16906479

[B39] Rothwell NJ Stock MJ Influence of clenbuterol on energy-balance, thermogenesis and body-composition in lean and genetically-obese Zucker rats Int J Obesity 1987 11 641 647 3440683

[B40] Navegantes LCC Resano NMZ Baviera AM Migliorini RH Kettelhut IC CL 316,243, a selective b3-adrenergic agonist, inhibits protein breakdown in rat skeletal muscle Pflugers Arch 2006 451 617 624 10.1007/s00424-005-1496-1 16091956

[B41] Bezaire V Spriet LL Campbell S Sabet N Gerrits M Bonen A Harper ME Constitutive UCP3 overexpression at physiological levels increases mouse skeletal muscle capacity for fatty acid transport and oxidation FASEB J 2005 19 977 980 15814607 10.1096/fj.04-2765fje

[B42] Nedergaard J Golozoubova V Matthias A Asadi A Jacobsson A Cannon B UCP1: the only protein able to mediate adaptive non-shivering thermogenesis and metabolic inefficiency Biochim Biophys Acta 2001 1504 82 106 10.1016/S0005-2728(00)00247-4 11239487

[B43] Romero MM Fernández-López JA Esteve M Alemany M Oleoyl-estrone inhibits lipogenic, but maintains thermogenic gene expression of brown adipose tissue in overweight rats Biosci Reports 2009 29 237 243 10.1042/BSR20080089 18828761

[B44] Remesar X Fernández-López JA Blay MT Savall P Salas A Díaz-Silva M Esteve M Grasa MM Alemany M Effect of oral oleoyl-estrone on adipose tissue composition in male rats Int J Obesity 2002 26 1092 1102 10.1038/sj.ijo.0802056 12119575

[B45] Collins S Cao W Robidoux J Learning new tricks from old dogs: β-adrenergic receptors teach new lessons on firing up adipose tissue metabolism Mol Endocrinol 2004 18 2123 2131 10.1210/me.2004-0193 15243132

[B46] Cinti S The adipose organ Prostagl Leukotrien Essent Fatty Acids 2005 73 9 15 10.1016/j.plefa.2005.04.010 15936182

[B47] Rajala MW Scherer PE The adipocyte - At the crossroads of energy homeostasis, inflammation, and atherosclerosis Endocrinology 2003 144 3765 3773 10.1210/en.2003-0580 12933646

