# Supplementary material for: Comparative effects of oleoyl-estrone and a specific β3-adrenergic agonist (CL316, 243) on the expression of genes involved in energy metabolism of rat white adipose tissue
Source: Nutr Metab (Lond). 2010 Feb 25;7:15. doi: 10.1186/1743-7075-7-15 (PMC2841192; doi:10.1186/1743-7075-7-15)
Supplement: Additional file 1 — WAT GENE EXPRESSIONS. content: Table S1 - Primers used in the analysis of gene expression. Table S2. Whole inguinal subcutaneous WAT content of specific gene mRNAs of rats overweight male treated 10 days with OE and B3A. Table S3. Whole epididymal WAT content of specific gene mRNAs of rats overweight male treated 10 days with OE and B3A. Table S4. Whole retroperitoneal WAT content of specific gene mRNAs of rats overweight male treated 10 days with OE and B3A. Table S5. Combined inguinal subcutaneous. epididymal and retroperitoneal WAT content of specific gene mRNAs of rats overweight male treated 10 days with OE and B3A. [file 1743-7075-7-15-S1.PDF]

SUPPLEMENTAL TABLES

Table S1 - Primers used in the analysis of gene expression

gene	primer sequence (5'>3')	primer sequence (3'>5')	size (bp)	Genebank GI
β_1-adrenoreceptor	TTCAACTGGCTGGGCTACGC	CAAAGCAGGCGCTGGAAA	92	6978458
β_2-adrenoreceptor	GGACAGACTACACAGGGGAGCA	CCAGGGGCTTCCTCACAAA	82	55926226
β_3-adrenoreceptor	CAGGCAGAACTCACCGCTCA	TCCAGAAGTCAGGCTCCTTGC	102	6978462
Phosphodiesterase 3B	GCCAGGTGTGCATCAAATTAGC	CCAGGGTTGCTTCTTCATCTCC	123	8393928
PPAR-α	TTCAATGCCCTCGAACTGGA	GCACAATCCCCTCCTGCAAC	124	6981381
PPAR-δ/β	CCAGCCATAACGCACCCTTC	TTCCACACCAGGCCCTTCTC	73	6981383
PPAR-γ_1	CACTTTCTGACCGGACTGTGTG	AAGTTGGTGGGCCAGAATGG	144	148747595
PPAR-γ_2	TCGCTGATGCACTGCCTATGA	CGAAGTTGGTGGGCCAGAAT	81	6981385
insulin receptor	TTGCTGAGGTGGGAGCCCTA	GCCCGTCAAACTCTGTCACG	84	8393620
IRS-1	AATGAGGGCAGCTCCCCAAG	GGTCCTGGTTGTGAATCGTGAA	198	6981105
insulin degrading enzyme	TGGACGCACCAAGGAGACAT	TGGCGCTTCGGAAAGGTTTA	116	6981075
GLUT4	CTTGATGACGGTGGCTCTGC	CACAATGAACCAGGGGATGG	127	6980957
SREBP1c	AAAACCAGCCTCCCCAGAGC	CCAGTCCCCATCCACGAAGA	153	109490841
acetyl-CoA carboxylase 1	AGGAAGATGGTGTCCGCTCTG	GGGGAGATGTGCTGGGTCAT	145	11559961
fatty acid synthase	CTTGGGTGCCGATTACAACC	GCCCTCCCGTACACTCACTC	163	8394157
lipoprotein lipase	GAAGGGGCTTGGAGATGTGG	TGCCTTGCTGGGGTTTTCTT	103	148747493
adiponutrin	GTGTGCCCGAATGACCATGT	GCCTTGGGGTTTGTGGAGAG	112	144226244
hormone- sensitive lipase	CAAGCCCCATAAGACCCCATT	CCGTAAGTCGCCCAGAATCC	93	6981163
adipocyte TAG lipase	GTGTGCCCGAATGACCATGT	GCCTTGGGGTTTGTGGAGAG	138	144226244
carnitine-palmitoleoyl trans.	GTGCTGGAGGTGGCTTTGGT	TGCTTGACGGATGTGGTTCC	152	6978702
fatty acid binding protein 4	CCTTTGTGGGGACCTGGAAA	TGACCGGATGACGACCAAGT	152	16758093
fatty acid transp. protein 1	GTGCGACAGATTGGCGAGTT	TGCGTGAGGATACGGCTGTT	106	50054323
UCP 1	CCTCTCCGGTGGATGTGGTAAA	CGCAGAAAAGAAGGCGCAAA	142	6981691
UCP 2	CTGGCGGTGGTCGGAGATAC	TTCGGGCAACATTGGGAGAG	108	148747398
UCP 3	GAATGGTGAGGAGGGGCTGT	TGGTTCCTTTGCTGCCTGTG	107	48675842
TNF-α	GGCTCCCTCTCATCAGTTCCA	CGCTTGGTGGTTTGCTACGA	104	82524821
leptin	CGGTTCCTGTGGCTTTGGT	CCGACTGCGTGTGTGAAATG	130	6981147
resistin	TCATGCCCAGAACCGAGTTG	CAGCCCCAGGACAAGGAAGA	109	21426804
adiponectin	GGAGACGCAGGTGTTCTTGG	AGCCCTACGCTGAATGCTGA	152	62990185
visfatin	TCTGGAAATCCGCTCGACAC	CACTCCGTCCCCTTGAATGA	129	55741461
ARBP (housekeeping gene)	CCCTTCTCCTTCGGGCTGAT	TGAGGCAACAGTCGGGTAGC	62	11693175

TABLE S2. Whole inguinal subcutaneous WAT content of specific gene mRNAs of rats overweight male treated 10 days with OE and B3A

gene	units	control	OE-treated	B3A-treated	OE+B3A-treated
β_1-adrenoreceptor	amol	232 ± 35	117 ± 16	341 ± 99	197 ± 23
β_2-adrenoreceptor	fmol	1.39 ± 0.17	0.77 ± 0.062	1.74 ± 0.41	1.35 ± 0.12
β_3-adrenoreceptor	fmol	1.57 ± 0.27	1.26 ± 0.19	6.30 ± 1.75	3.21 ± 0.56
Phosphodiesterase 3B	amol	148 ± 8	131 ± 28	817 ± 227	614 ± 203
PPAR-α	amol	78 ± 9	26 ± 5	223 ± 49	80 ± 20
PPAR-δ/β	amol	393 ± 89	113 ± 23	427 ± 175	218 ± 66
PPAR-γ_1	amol	247 ± 55	133 ± 34	414 ± 142	425 ± 87
PPAR-γ_2	fmol	0.488 ± 0.107	0.231 ± 0.070	1.93 ± 0.66	1.58 ± 0.34
insulin receptor	amol	172 ± 35	51 ± 11	581 ± 210	145 ± 35
IRS-1	amol	135 ± 29	36 ± 14	338 ± 120	142 ± 44
insulin degrading enzyme	fmol	0.525 ± 0.061	0.221 ± 0.061	2.03 ± 0.64	0.782 ± 0.249
GLUT4	fmol	2.98 ± 0.22	1.25 ± 0.37	9.27 ± 2.72	3.95 ± 0.91
SREBP1c	fmol	5.89 ± 0.57	2.41 ± 0.47	18.3 ± 3.8	3.55 ± 1.53
acetyl-CoA carboxylase 1	fmol	1.56 ± 0.21	0.455 ± 0.102	12.6 ± 4.7	3.50 ± 1.31
fatty acid synthase	fmol	27.4 ± 8.5	5.39 ± 1.35	478 ± 222	39.9 ± 19.8
lipoprotein lipase	fmol	39.2 ± 7.8	16.0 ± 5.0	164 ± 56	65.8 ± 17.2
adiponutrin	fmol	1.30 ± 0.26	0.072 ± 0.022	17.2 ± 8.6	1.56 ± 0.63
hormone- sensitive lipase	fmol	13.2 ± 1.3	9.54 ± 1.03	43.2 ± 12.0	36.1 ± 10.0
adipocyte TAG lipase	fmol	33.4 ± 3.2	38.7 ± 5.6	151 ± 33	113 ± 28
carnitine-palmitoleoyl trans.	fmol	0.711 ± 0.082	0.826 ± 0.354	2.73 ± 0.91	2.85 ± 0.48
fatty acid binding protein 4	fmol	229 ± 44	333 ± 39	1366 ± 323	1149 ± 139
fatty acid transp. protein 1	fmol	0.998 ± 0.173	0.765 ± 0.214	4.93 ± 0.50	3.20 ± 1.17
UCP 1	fmol	1.09 ± 1.09	0.002 ± 0.000	3.58 ± 1.32	0.156 ± 0.090
UCP 2	fmol	5.85 ± 0.23	2.24 ± 0.60	18.4 ± 12.30	6.98 ± 2.65
UCP 3	fmol	0.389 ± 0.090	0.130 ± 0.054	1.41 ± 0.44	0.630 ± 0.213
TNF-α	amol	28 ± 9	7 ± 2	28 ± 8	27 ± 13
leptin	fmol	4.99 ± 1.32	1.52 ± 0.53	7.03 ± 2.65	2.08 ± 0.82
resistin	fmol	34.0 ± 8.9	3.95 ± 1.34	232 ± 58	96.2 ± 26.3
adiponectin	fmol	54.6 ± 10.3	32.8 ± 8.6	206 ± 66	153 ± 37
visfatin	fmol	1.86 ± 0.33	0.717 ± 0.238	8.02 ± 1.11	3.62 ± 1.21

The data correspond to the mean ± sem of 6 different animals. The results of the statistical analysis (two-way ANOVA) of these data are presented in Table 3 of the main text.

TABLE S3. Whole epididymal WAT content of specific gene mRNAs of rats overweight male treated 10 days with OE and B3A

gene	units	control	OE-treated	B3A-treated	OE+B3A-treated
β_1-adrenoreceptor	amol	656 ± 13	501 ± 66	546 ± 111	385 ± 11
β_2-adrenoreceptor	fmol	1.15 ± 0.12	1.28 ± 0.11	1.60 ± 0.10	1.28 ± 0.10
β_3-adrenoreceptor	fmol	10.6 ± 1.9	8.33 ± 1.32	16.3 ± 5.9	14.4 ± 0.2
Phosphodiesterase 3B	fmol	0.510 ± 0.099	0.553 ± 0.095	2.83 ± 0.85	2.01 ± 0.07
PPAR-α	amol	87 ± 12	54 ± 12	203 ± 59	344 ± 71
PPAR-δ/β	amol	341 ± 69	138 ± 29	907 ± 276	664 ± 160
PPAR-γ_1	amol	369 ± 70	165 ± 36	736 ± 215	389 ± 58
PPAR-γ_2	fmol	1.20 ± 0.25	0.786 ± 0.217	2.65 ± 0.78	2.16 ± 0.27
insulin receptor	amol	241 ± 77	241 ± 42	623 ± 157	526 ± 35
IRS-1	amol	128 ± 33	132 ± 32	207 ± 55	180 ± 14
insulin degrading enzyme	fmol	0.840 ± 0.219	0.575 ± 0.067	1.50 ± 0.48	1.30 ± 0.21
GLUT4	fmol	5.14 ± 0.34	1.73 ± 0.19	8.95 ± 0.84	5.23 ± 0.49
SREBP1c	fmol	6.33 ± 1.10	3.06 ± 0.59	12.7 ± 3.0	12.4 ± 1.4
acetyl-CoA carboxylase 1	fmol	2.44 ± 0.32	0.873 ± 0.188	7.50 ± 2.21	7.00 ± 0.58
fatty acid synthase	fmol	31.1 ± 7.7	5.61 ± 0.77	99.3 ± 33.3	84.5 ± 16.3
lipoprotein lipase	fmol	62.9 ± 10.4	21.7 ± 5.4	161 ± 55	58.0 ± 6.9
adiponutrin	fmol	7.07 ± 0.31	0.462 ± 0.101	14.8 ± 6.8	2.19 ± 0.41
hormone- sensitive lipase	fmol	43.9 ± 6.8	41.4 ± 3.0	82.6 ± 24.7	82.5 ± 14.5
adipocyte TAG lipase	fmol	87.9 ± 8.1	137 ± 21	328 ± 88	265 ± 46
carnitine-palmitoleoyl trans.	fmol	2.58 ± 0.56	3.57 ± 0.59	10.4 ± 3.2	12.6 ± 3.3
fatty acid binding protein 4	pmol	0.828 ± 0.110	0.939 ± 0.073	2.29 ± 0.72	1.67 ± 0.31
fatty acid transp. protein 1	fmol	1.19 ± 0.28	1.00 ± 0.20	5.75 ± 1.59	6.77 ± 0.22
UCP 1	fmol	0.037 ± 0.014	0.019 ± 0.006	13.8 ± 4.3	11.1 ± 4.2
UCP 2	fmol	12.7 ± 1.45	6.75 ± 0.62	15.6 ± 4.2	18.4 ± 5.1
UCP 3	fmol	0.510 ± 0.121	0.457 ± 0.117	2.39 ± 0.85	1.28 ± 0.13
TNF-α	amol	34 ± 3	49 ± 4	52 ± 13	49 ± 5
leptin	fmol	11.7 ± 3.5	2.84 ± 0.60	14.7 ± 3.5	2.76 ± 0.77
resistin	fmol	65.3 ± 13.8	21.8 ± 5.0	395 ± 27	237 ± 54
adiponectin	fmol	257 ± 29	144 ± 18	388 ± 99	247 ± 22
visfatin	fmol	1.58 ± 0.36	1.25 ± 0.30	7.10 ± 2.58	6.46 ± 0.69

The data correspond to the mean ± sem of 6 different animals. The results of the statistical analysis (two-way ANOVA) of these data are presented in Table 3 of the main text.

TABLE S4. Whole retroperitoneal WAT content of specific gene mRNAs of rats overweight male treated 10 days with OE and B3A

gene	units	control	OE-treated	B3A-treated	OE+B3A-treated
β_1-adrenoreceptor	amol	863 ± 223	442 ± 90	390 ± 22	261 ± 16
β_2-adrenoreceptor	fmol	1.19 ± 0.25	1.14 ± 0.19	1.10 ± 0.11	0.967 ± 0.114
β_3-adrenoreceptor	fmol	6.46 ± 1.89	6.86 ± 0.28	12.8 ± 2.0	8.29 ± 1.22
Phosphodiesterase 3B	fmol	0.495 ± 0.117	0.473 ± 0.107	1.60 ± 0.22	1.16 ± 0.270
PPAR-α	amol	113 ± 59	71 ± 10	470 ± 9	179 ± 67
PPAR-δ/β	amol	525 ± 220	245 ± 52	839 ± 257	306± 26
PPAR-γ_1	amol	409 ± 83	404 ± 56	394 ± 97	260 ± 50
PPAR-γ_2	fmol	1.10 ± 0.26	1.32 ± 0.22	1.98 ± 0.46	1.38 ± 0.22
insulin receptor	amol	283 ± 127	117 ± 21	264 ± 70	181 ± 34
IRS-1	amol	181 ± 77	125 ± 21	240 ± 77	164 ± 10
insulin degrading enzyme	fmol	0.715 ± 0.213	0.563 ± 0.087	1.04 ± 0.15	0.623 ± 0.067
GLUT4	fmol	11.1 ± 2.4	3.69 ± 0.70	11.4 ± 1.7	5.49 ± 0.94
SREBP1c	fmol	14.8 ± 3.1	5.78 ± 1.33	13.5 ± 3.4	10.3 ± 0.7
acetyl-CoA carboxylase 1	fmol	4.72 ± 0.97	1.54 ± 0.19	11.4 ± 1.3	5.92 ± 0.64
fatty acid synthase	fmol	122 ± 41	24.1 ± 7.0	181 ± 29	66.3 ± 19.9
lipoprotein lipase	fmol	88.4 ± 20.5	63.9 ± 4.4	145 ± 34	72.6 ± 14.8
adiponutrin	fmol	8.97 ± 1.60	1.30 ± 0.35	13.60 ± 2.04	0.911 ± 0.185
hormone- sensitive lipase	fmol	47.0 ± 9.8	57.6 ± 8.8	80.4 ± 12.9	52.4 ± 10.5
adipocyte TAG lipase	fmol	152 ± 33	161 ± 32	204 ± 36	226 ± 16
carnitine-palmitoleoyl trans.	fmol	4.07 ± 1.14	3.59 ± 0.91	14.38 ± 3.32	10.8 ± 0.5
fatty acid binding protein 4	pmol	1.76 ± 0.31	1.32 ± 0.178	1.98 ± 0.23	1.42 ± 0.09
fatty acid transp. protein 1	fmol	2.43 ± 0.49	3.69 ± 0.85	10.0 ± 2.2	7.99 ± 1.36
UCP 1	fmol	0.341 ± 0.144	12.1 ± 7.3	64.2 ± 25.4	88.7 ± 44.7
UCP 2	fmol	7.21 ± 1.60	3.65 ± 0.52	11.9 ± 1.6	7.50 ± 0.97
UCP 3	fmol	0.660 ± 0.189	0.536 ± 0.080	2.29 ± 0.70	1.86 ± 0.06
TNF-α	amol	21 ± 5	14 ± 3	47 ± 5	32 ± 3
leptin	fmol	19.1 ± 4.0	7.56 ± 1.33	4.53 ± 1.86	0.785 ± 0.115
resistin	fmol	61.2 ± 11.5	30.3 ± 5.3	321± 54	190 ± 45
adiponectin	fmol	142 ± 25	150 ± 21	205 ± 30	162 ± 20
visfatin	fmol	2.11 ± 0.59	2.23 ± 0.55	4.41 ± 1.09	5.06 ± 0.74

The data correspond to the mean ± sem of 6 different animals. The results of the statistical analysis (two-way ANOVA) of these data are presented in Table 3 of the main text.

TABLE S5. Combined inguinal subcutaneous. epididymal and retroperitoneal WAT content of specific gene mRNAs of rats overweight male treated 10 days with OE and B3A

gene	units	control	OE-treated	B3A-treated	OE+B3A-treated
β_1-adrenoreceptor	fmol	1.75 ± 0.25	1.06 ± 0.11	1.28 ± 0.16	0.843 ± 0.019
β_2-adrenoreceptor	fmol	3.73 ± 0.34	3.19 ± 0.28	4.44 ± 0.44	3.60 ± 0.16
β_3-adrenoreceptor	fmol	18.6 ± 1.5	16.5 ± 1.5	35.5 ± 8.8	25.9 ± 0.6
Phosphodiesterase 3B	fmol	1.15 ± 0.14	1.16 ± 0.12	5.25 ± 0.88	3.79 ± 0.33
PPAR-α	amol	279 ± 33	151 ± 20	896 ± 48	604 ± 102
PPAR-δ/β	fmol	1.26 ± 0.25	0.50 ± 0.06	2.17 ± 0.56	1.19 ± 0.20
PPAR-γ_1	fmol	1.03 ± 0.07	0.702 ± 0.062	1.54 ± 0.34	1.07 ± 0.09
PPAR-γ_2	fmol	2.78 ± 0.32	2.33 ± 0.38	6.57 ± 1.37	5.11 ± 0.52
insulin receptor	fmol	0.695 ± 0.144	0.409 ± 0.045	1.47 ± 0.15	0.852 ± 0.053
IRS-1	amol	444 ± 94	293 ± 32	784 ± 203	486 ± 47
insulin degrading enzyme	fmol	2.08 ± 0.19	1.36 ± 0.15	4.57 ± 0.67	2.70 ± 0.40
GLUT4	fmol	19.2 ± 1.5	6.67 ± 1.02	29.6 ± 4.9	14.7 ± 1.5
SREBP1c	fmol	27.0 ± 2.9	11.2 ± 1.1	44.4 ± 3.8	26.2 ± 1.7
acetyl-CoA carboxylase 1	fmol	8.73 ± 1.10	2.87 ± 0.32	31.5 ± 3.95	16.4 ± 1.7
fatty acid synthase	fmol	181 ± 47	35.1 ± 6.6	759 ± 214	191 ± 21
lipoprotein lipase	fmol	191 ± 23	102 ± 11	471 ± 97	196 ± 25
adiponutrin	fmol	17.3 ± 1.4	1.84 ± 0.31	45.6 ± 9.8	4.66 ± 0.54
hormone- sensitive lipase	fmol	104 ± 8	109 ± 9	206 ± 43	171 ± 21
adipocyte TAG lipase	fmol	274± 31	337 ± 41	683 ± 91	604 ± 44
carnitine-palmitoleoyl trans.	fmol	7.37 ± 0.95	7.99 ± 1.22	27.5 ± 3.2	26.3 ± 3.6
fatty acid binding protein 4	pmol	2.82 ± 0.35	2.59 ± 0.14	5.63 ± 0.70	4.24 ± 0.32
fatty acid transp. protein 1	fmol	4.62 ± 0.52	5.46 ± 0.98	20.7 ± 2.6	18.0 ± 1.2
UCP 1	fmol	1.47 ± 0.22	12.1 ± 6.0	81.6 ± 18.6	99.9 ± 32.0
UCP 2	fmol	25.8 ± 2.2	12.6 ± 0.9	45.8 ± 6.6	32.9 ± 7.0
UCP 3	fmol	1.56 ± 0.09	1.12 ± 0.19	6.09 ± 1.14	3.77 ± 0.27
TNF-α	amol	83 ± 12	70 ± 4	127 ± 12	108 ± 13
leptin	fmol	35.8 ± 4.2	11.9 ± 2.0	26.3 ± 6.3	5.6 ± 1.2
resistin	fmol	160 ± 13	56.1 ± 6.5	948 ± 112	523 ± 56
adiponectin	fmol	453 ± 26	326 ± 37	799 ± 175	563 ± 53
visfatin	fmol	5.55 ± 0.59	4.20 ± 0.52	19.5 ± 2.3	15.1 ± 1.7

The data correspond to the mean ± sem of 6 different animals. The results of the statistical analysis (two-way ANOVA) of these data are presented in Table 3 of the main text.
